# Prevalence of Her2-neu status and its clinicopathological association in newly diagnosed gastric cancer patients

**DOI:** 10.1186/s12885-022-10206-1

**Published:** 2022-11-01

**Authors:** Joseph Kattan, Fady el Karak, Fadi Farhat, Dany Abi Gerges, Walid Mokaddem, Georges Chahine, Saad Khairallah, Najla Fakhruddin, Jawad Makarem, Fadi Nasr

**Affiliations:** 1 Hôtel-Dieu de France University Hospital, Beirut, Lebanon; 2 Saint-Joseph University, Beirut, Lebanon; 3 Department of Hematology and Oncology, Hammoud Hospital University Medical Center, Saida, Lebanon; 4 Department of Hematology and Oncology, Middle East Institute of Health, Bsalim, Lebanon; 5 Department of Hematology and Oncology, Haykal Hospital, Tripoli, Lebanon; 6 National Institute of Pathology, Baabda, Lebanon; 7 Department of Pathology, Hammoud Hospital, Saida, Lebanon; 8 Department of Hematology and Oncology, Ain W Zain Hospital, Ain W Zain, Lebanon; 9 Department of Hematology and Oncology, Mount Lebanon Hospital, Beirut, Lebanon

**Keywords:** Gastric cancer, HER2-neu, Immunohistochemistry (IHC), *In-situ* hybridization (ISH), Prevalence

## Abstract

**Background:**

This study aimed to report the prevalence of HER2-neu in newly diagnosed early or metastatic gastric cancer (GC) patients, to determine the percentage of patients achieving various IHC scores correlating with the ISH results and to establish a database for GC patients in Lebanon.

**Methods:**

This was a national, multicenter, descriptive and cross-sectional study in patients with histologically confirmed early or metastatic GC newly diagnosed. All eligible patients underwent the IHC and ISH tests in a central laboratory. Demographics, medical history and histopathology data were collected.

**Results:**

One hundred fifty-seven patients were included (mean age at diagnosis: 63 ± 14.1 years) during a 3.5 year period. The prevalence of HER2-neu over expression was 21% (95% CI: 15.3–27.4) using ICH and ISH. Agreement between IHC and ISH results was significantly substantial (kappa = 0.681; *p*-value < 0.001). Over expressed HER2-neu status was significantly associated with high ECOG performance status only.

**Conclusions:**

The prevalence of HER2-neu over expression in newly diagnosed early or metastatic GC patients seemed to be high in Lebanon. The database generated allows to monitor trends in the epidemiology and management of GC.

## Introduction

Gastric cancer (GC) is the fourth most common cause of cancer-related death in the world. More than one million cases of GC were diagnosed in 2020 globally, and 769,000 deaths were attributed to GC in the same year [1]. The prevalence of GC is two-fold higher in men compared to women and varies geographically, with a much higher prevalence in Eastern Asian and Eastern European countries [1]. In Lebanon, GC is responsible for approximately 2.2% of all cancer cases, 2.4% (incidence rate of 6.4 per 100,000) of all male cancers and 2% (incidence rate of 5.2 per 100,000) of all female cancers based on the 2016 National Cancer Registry estimates [2]. A recently published study from Lebanon found that GC accounted for 15.5% of all gastrointestinal cancer cases between 2001 and 2015 [3].

Most cases of GC will present with advanced disease at diagnosis and have a poor outcome [4, 5]. GC risk factors have been identified and include gender [6] socioeconomic status [7], dietary factors such as salt intake and tobacco, coffee and alcohol consumption [8–10], certain autoimmune disorders [11], hereditary and environmental factors [12, 13], *Helicobacter pylori* infection [9, 14, 15], and gastric surgery [16]. Several factors can predict poor prognosis in GC, such as older age, larger tumor size, lymphovascular invasion, and lymph node metastasis [17]. The human epidermal growth factor receptor 2 (HER2-neu, aka HER2) is overexpressed in up to 22% of GC patients [18], and is currently one of the three available biomarkers of response to targeted therapy in GC. HER2-neu has also emerged as a prognostic factor for poor outcomes, worse survival and a more aggressive disease in GC [19–21].

As for treatment, trastuzumab (Herceptin®, F. Hoffmann-La Roche Ltd, Basel, Switzerland), is a humanized monoclonal antibody directed against HER2 that was approved in 2010 for use in metastatic GC tumors with HER2 overexpression in combination with chemotherapy. Trastuzumab remains the only approved treatment for GC that overexpresses HER2-neu and its use has led to significant improvements in survival [22, 23]. Other novel HER2-targeting therapies are currently under trial and are expected to be invaluable in facing the inevitable emergence of resistance to trastuzumab. Current practice guidelines recommend that HER2 status be assessed at diagnosis in all metastatic GC patients to provide ideal patient management [24]. HER2 status can be assessed through different modalities, such as immunohistochemistry (IHC), *in-situ* hybridization (ISH), and Next-Generation sequencing (NGS). IHC, the most commonly used technique, semi-quantitively assesses the expression of the HER2 protein on the surface of tumor cells. HER2 protein levels can then be scored on a scale of 0–3 + and determined to be negative (scores of 0 and 1 +), equivocal (score of 2 +), or over-expressed (score of 3 +). Alternatively, ISH employs DNA probes to reflect the level of HER2 gene amplification. DNA probes can be fluorescently labeled (fluorescence ISH (FISH)) or DNP-labelled coupled with centromeric probes with chromogenic detection (Dual SISH (BDISH). Based on this, tumor overexpression of HER2 can be established through high protein levels (IHC 3 +) or HER2 gene amplification (FISH/Dual SISH positive). The application of IHC as the first-line testing method allows the identification of patients with HER2-over expressed disease (3 +) who are candidates for trastuzumab, and the exclusion of patients with HER2-negative disease (0/1 +). In parallel, IHC 2 + tumor samples should be retested for amplification of the HER2 gene by FISH or Dual SISH to ensure that all patients who may benefit from trastuzumab are identified [24].

Given these considerations, an epidemiological study was designed to assess the prevalence of HER2-neu overexpression in newly diagnosed early or metastatic GC patients residing in Lebanon. It also aimed to determine the percentage of patients achieving various IHC scores correlating with the ISH results and to establish a database for GC patients in Lebanon in terms of referral and treatment trends of GC, taking into consideration several prognostic factors, mainly HER2-neu status and disease stage.

## Methods

### Study design and study population

This national multicenter, descriptive, cross-sectional cohort study was conducted in Lebanon. Patients aged over 18 years with newly diagnosed and histologically confirmed early or metastatic GC were enrolled after having signed an informed consent. Exclusion criteria included the participation in interventional or observational studies within the last 30 days, pregnancy, lactation and age below 18 years old.

### Study procedures

All eligible patients underwent both the IHC and ISH (FISH or BDFISH) tests in a central laboratory. All paraffin blocks for testing were sent from the medical centers to the central laboratory using a courier service after completion of a request form by the investigator. The form was sent to the central laboratory along with the paraffin block for testing. The result of the HER2-testing was directly sent to the investigator by the central laboratory using the same courier service and the delivery was done the next day.

### Data sources

The patients’ socio-demographic data (date of birth, gender, marital status, residency area, occupation) risk factors (weight, height and body mass index [BMI]), smoking habits, consumption of fatty foods and anti-oxidants and family history of GC) were collected on a case report form. The medical history of the patients were also recorded and included the following information: date of first GC symptom, date of diagnosis, concomitant diseases (especially *Helicobacter pylori* infection, gastroesophageal reflux disease [GERD], hereditary non-polyposis colorectal cancer [HNPCC] and pernicious anemia), history of cardiac or pulmonary diseases, type of gastric surgery, site of cancer, biopsy, x-ray and endoscopy results, blood sampling results (complete blood count, vitamin B12 deficiency and carcinoembryonic antigen (CEA) 19–9 status), HER2-neu status, Tumor, Node, Metastasis (TNM) staging, sites of metastasis, Eastern Cooperative Oncology Group (ECOG) performance status (PS), referral system and therapeutic decision.

### Sample size and statistical analysis

The sample size was computed using the formula (*n* = z^2^pq/d^2^) where, the sample size (n) is equal to the squared z-value of desired significance level (90%) multiplied by the expected prevalence of positive HER2-neu among GC cases (18.8% according to Xie et al*.* [25] and the expected absence of positive HER2-neu (81.2%) divided by the squared acceptable range for the prevalence under study (d = 5%). Based on these calculations, a minimum sample of 170 participants was required.

Patients with complete outcome data were included in the final analysis. Data were analyzed using IBM SPSS, version 20.0 for Windows release (IBM Corp. Released 2011. IBM SPSS Statistics for Windows, Version 20.0. Armonk, NY: IBM Corp.). Data were summarized using means, standard deviations, medians and interquartile ranges for continuous variables, and frequency distribution for categorical variables. The association of HER2-neu status with demographic, clinical and pathological variables was examined using the Pearson chi-square or Fisher’s exact test as appropriate for categorical variables, and Student’s *t*-test for continuous variables. The association of HER2-neu status with categorical variables of more than two categories was studied using logistic regression. Receiver Operating Characteristic (ROC) curve was performed to check true positive rate against the false positive rate taking IHC as a screening test and ISH as the gold standard for diagnosis in the Lebanese population. Area under the curve (AUC) was the method used to test the accuracy of the test: an area of 1 represented a perfect test while an area of 0.5 represented a worthless test. A test was considered a good screening test if AUC was ≥ 0.8. Additional analysis was performed to assess the level of agreement between the IHC and ISH results in predicating HER2-neu status using Cohen’s kappa coefficient according to Landis and Kosh’s classification, which is universally accepted (Table 1) [26]. A two-sided *p*-value < 0.05 was considered statistically significant.

**Table 1:** Interpretation of kappa according to Landis and Kosh [27]

**Kappa**	**Agreement**
< 0	Less than chance agreement (poor)
0.01–0.20	Slight agreement
0.21–0.40	Fair agreement
0.41–0.60	Moderate agreement
0.61–0.80	Substantial agreement
0.81–0.99	Almost perfect agreement

### Ethics consideration

This study was conducted in accordance with the Declaration of Helsinki and Good Pharmacoepidemiology Practice (GPP). It was also approved by the ethics committees of each of the participating centers (ethics committee named after participating centers), which were: Ain Wa Zein Hospital, Bellevue Medical Center, Middle East Institute of Health, Haykel Hospital, Hotel Dieu De France, Hammoud University Hospital, Mount Lebanon Hospital, Beirut Governmental University Hospital- Rafic Hariri Governmental Hospital, and Saint Joseph University. All participants completed an informed consent form before their enrollment. All data were analyzed anonymously.

## Results

### Socio-demographic characteristics and risk factors

Between 12 April 2011 and 15 September 2014, a total of 157 newly diagnosed patients (88 [56.1%] men) with histologically confirmed early or metastatic GC were enrolled from nine sites from all Lebanese governorates. The mean age was 63.0 ± 14.1 years and the majority (*n* = 149; 94.9%) of the patients were Lebanese with 82 (52.3%) patients living in Mount Lebanon and 88 (56.1%) in the urban areas across the country. Overall, 129 (82.8%) patients were married, 84 (53.5%) were not employed. As for the risk factors, 51 (32.5%) were smokers and the majority of the patients (*n* = 64, 40.8%) had a normal healthy weight (Table 2).

**Table 2:** Socio-demographic characteristics and risk factors of the patients (*N* = 157)

**Characteristics**	
**Socio-demographics**	
Gender, n(%)	
Men	88 (56.1%)
Women	69 (43.9%)
Age at diagnosis (years)	
Mean ± standard deviation	63.0 ± 14.1
Range: minimum; maximum	29; 89
Residency area, n(%)	
Rural	61 (38.9%)
Urban	88 (56.1%)
Missing	8 (5.1%)
Geographical distribution, n(%)	
Beirut	17 (10.8%)
Bekaa	24 (15.3%)
Mount Lebanon	52 (33.1%)
Nabatiyeh	2 (1.3%)
North	28 (17.8%)
South	20 (12.7%)
**Risk factors**	
Smoking, n(%)	
No	90 (57.3%)
Yes	51 (32.5%)
Missing	16 (10.2%)
Alcohol consumption, n(%)	
No	90 (81.9%)
Yes	51 (7.7%)
Missing	16 (10.3%)
Fatty food consumption, n(%)	
No	123 (78.3%)
Yes	28 (17.8%)
Missing	6 (3.8%)
Antioxidant consumption, n(%)	
No	149 (95.5%)
Yes	2 (1.3%)
Missing	5 (3.2%)
BMI categories, n(%)	
Underweight (< 18 kg/m^2^)	8 (5.1%)
Normal healthy weight (18.5–24.9 kg/m^2^)	64 (40.8%)
Overweight (25–29.9 kg/m^2^)	43 (27.4%)
Obese (> 30 kg/m^2^)	21 (13.4%)
Missing	21 (13.4%)
Family history, n(%)	
No	141 (89.8%)
Yes	4 (2.5%)
Missing	12 (7.6%)

*Abbreviations*: *BMI* Body mass index

### Patients’ medical history

History of digestive system diseases showed that 8 (5.1%) patients had GERD, 19 (12.1%) had *Helicobacter pylori* infections and 7 (4.5%) had pernicious anemia. No patients were reported to be previously suffering from HNPCC. Cardiac diseases were reported in 38 (24.2%) patients and pulmonary diseases in 5 (3.2%) patients. The participants also suffered from other diseases such as diabetes mellitus (*n* = 23, 14.6%) and hypertension (*n* = 20, 12.7%).

### Gastric cancer characteristics

Most of the GC cases occurred in the cardia (*n* = 45, 28.7%), followed by the antrum (*n* = 37, 23.6%), fundus (*n* = 22, 14%) and lesser curvature (*n* = 17, 10.8%). Other GC sites included: body of the stomach (*n* = 12, 7.6%), pylorus / antrum pylorus (*n* = 7, 4.5%), greater curvature (n = 6, 3.8%), posterior wall (*n* = 3, 1.9%), anterior wall and circle (*n* = 2, 1.3% each). Moreover, cancer started in the esophagus (*n* = 17, 10.8%) and the duodenum (*n* = 3, 1.9%). The most common PS was ECOG 1 (*n* = 102, 65%), followed by 2 (*n* = 35, 22.3%). Abnormal levels of hemoglobin were reported in 73 (47%) patients and lymphocyte count in 29 (18.5%) patients. As for tumor characterization, endoscopy showed that 111 (70.7%) GC cases were penetrating while only 15 (9.6%) were protruding. Also, Stage IIIB (*N* = 19; 12.1%) and Stage IV (*n* = 76; 48.4%) were the most common GC stages. The most common site of metastasis was the liver in 25 (34.0%) patients. The majority of patients had tubular carcinoma (59%) while only 34 patients (31.7%) had Signet-ring cell carcinoma (Table 3).

**Table 3:** Gastric cancer characteristics (*N* = 157)

**Characteristics**	
ECOG status, n(%)	
0	0 (0%)
1	102 (65.0%)
2	35 (22.3%)
3	15 (9.6%)
4	4 (2.5%)
Missing	1 (0.6%)
Endoscopy results, n(%)	
Penetrating	111 (70.7%)
Protruding	15 (9.6%)
Miscellaneous	6 (3.8%)
Superficial	3 (1.9%)
Linitis	3 (1.9%)
Not applicable	15 (9.6%)
Not done	4 (2.5%)
CT scan results, n(%)	
Coeliac lymphadenopathy	28 (17.8%)
Liver metastasis	19 (12.1%)
Other metastasis (bone, lung, ovarian, etc.)	69 (43.9%)
No metastasis	30 (19.1%)
Missing	11 (7.0%)
Chest x-ray results, n(%)	
Normal	18 (11.5%)
Central infiltration	2 (1.3%)
Other	8 (5.1%)
Missing	129 (82.2%)
Laboratory results, mean ± standard deviation	
CA 19–9 (U/mL)	1,122.00 ± 2,493.00
CEA (ng/ml)	91.43 ± 419.01
Hemoglobin (g/dL)	11.50 ± 2.20
Red blood cells count (10^12/L)	4.19 ± 0.76
White blood cells count (10^9/L)	7.30 ± 3.40
Platelets count (10^9/L)	334.00 ± 254.00
Lymphocytes count (%)	23.30 ± 12.90
Neutrophils count (%)	58.00 ± 21.50
Abnormal results, n(%)	
Hemoglobin	73 (46.5%)
Red blood cells	48 (30.6%)
Lymphocytes	29 (18.5%)
Neutrophils	26 (16.6%)
White blood cells	25 (15.9%)
Monocytes	19 (12.1%)
CA	14 (8.9%)
Eosinophils	14 (8.9%)
CEA	9 (5.7%)
Vitamin B12	6 (3.8%)
Basophils	5 (3.2%)
Gastric cancer stage, n(%)	
IA	3 (1.9%)
IB	2 (1.3%)
II	33 (21.0%)
IIIA	12 (7.6%)
IIIB	19 (12.1%)
IV	76 (48.4%)
Not applicable	12 (7.6%)
Sites of metastases, n(%)	
Bone	5 (3.2%)
Brain	1 (0.6%)
Liver	25 (15.9%)
Lung	5 (3.2%)
NA	2 (1.3%)
No metastasis	79 (50.3%)
Other	40 (25.5%)
Histology results, n(%)	
Hepatoid adenocarcinoma	1 (0.6%)
Mucinous adenocarcinoma	7 (4.5%)
Other	19 (11.5%)
Papillary adenocarcinoma	3 (1.9%)
Signet-ring cell carcinoma	34 (21.7%)
Tubular adenocarcinoma, moderately differentiated	44 (28.0%)
Tubular adenocarcinoma, well differentiated	11 (7.0%)
Tubular adenocarcinoma, poorly differentiated	38 (24.2%)
Undifferentiated carcinoma	1 (0.6%)

*Abbreviations*: *CA* Cancer antigen, *CEA* Carcinoembryonic antigen, *ECOG* Eastern Cooperative Oncology Group

### Disease management

A total of 55 (35%) patients underwent surgery. Referral was mainly done by a gastroenterologist (*n* = 46, 29.3%) and surgeon (*n* = 44, 28%). Neoadjuvant therapy was proposed to almost half of the operated patients (*n* = 26; 47.2%). Out of the 26 patients who received a neoadjuvant therapy, anthracycline-based triplets were given to 15 (58%) patients, docetaxel -based therapy was given to 9 (34.6%) patients. First-line trastuzumab was given to 10/79 (12.7%) patients.

### HER2-Neu status

IHC results showed that 10 (6.4%) patients had a HER2-over expressed disease (+ 3), 75 (47.7%) patients with HER2-negative disease (0/1 +) and 72 (45.9%) cases were equivocal (2 +). In parallel, ISH identified 33 (21.0%) HER2-over expressed patients and 111 (70.7%) HER2-negative patients; 13 (8.3%) patients’ results were non-contributive. Based on IHC and ISH results, 8 of 75 patients were found HER2-over expressed with a significantly substantial level of agreement between both techniques (kappa = 0.681; *p*-value < 0.001); the positive percent agreement was 66.67% and the negative percent agreement was 96.82%. IHC had a total of 72 equivocal results with 3 non-contributive ISH (Table 4). Considering ISH as the gold standard, 33 (21%) patients were found HER2-over expressed, with a HER2-neu prevalence of 21% (95% CI: 15.3–27.4) in the sample.

**Table 4:** Equivocal IHC results versus ISH results

		**ISH**		
		**Over expressed**	**Negative**	**Total**
		**n(%)**	**n(%)**	**n(%)**
**IHC**^**a**^	Equivocal	21 (30.4%)	48 (69.6%)	69 (100%)

*Abbreviations*: *IHC* Immunohistochemistry, *ISH In-situ* hybridization

^a^IHC had a total of 72 equivocal results with 3 non contributive ISH

Finally, bivariate analysis showed that ECOG PS was the only factor significantly associated with HER2 status as ECOG PS scores 1 to 3 were less frequent while score 4 was more frequent in the HER2-over expressed patients compared with HER2-negative patients (score 1: 13% versus 53.2%; score 2: 3.2% versus 17.5%; score 3: 3.2% versus 6.5%; score 4: 1.9% versus 0.8%; *p*-value = 0.018). Other factors such as age, gender, family history, dietary and lifestyle habits (smoking, alcohol consumption, antioxidants, fatty foods and BMI), stages, GERD, *Helicobacter pylori* infections, pernicious anemia, presence of metastasis and treatment modalities were not significantly different between HER2-over expressed and HER2-negative patients.

## Discussion

The prevalence of HER2-neu overexpression in GC was 21% in the present study, as determined using the HER-2 measurement procedure (ICH and ISH). This finding is aligned with the literature from Western and developed countries. Earlier screening data from the ToGA study showed an overall rate of 22.1% HER2-positivity, with 23.6% and 23.9% described in Europe and Asia, respectively [18]. A more recent large multinational study of close to 5000 GC patients reported an overall prevalence of 14.2% HER2-over expression [28]. When looking at rates from individual countries, the prevalence of HER2-positivity found in the present study was comparable to that of GC patients from France (20.2%), yet is superior to the rate of 12.8% and 12% described in Asia and Europe, respectively [28].

The variability of HER2 overexpression rates is evident in GC and incidence can vary from 4 to 53% [29]. A previous study retrospectively reported 16.2% HER2 overexpression in a population of GC patients from a single medical institution in Lebanon [30]. A lower rate of 10.3% HER2 overexpression was found in a prospective cohort study of 58 patients from a single Lebanese center [27]. Studies offering insight into HER2 prevalence among GC cases in the Middle East region in general, and Lebanon in particular, remain scarce [31, 32]. To the best of our knowledge, this study is the first multicentric effort to prospectively investigate the prevalence of HER2-neu overexpression in newly diagnosed early or metastatic GC patients residing in Lebanon specifically, as well as in Arab countries in general.

In our study, no significant association was found between HER2-overexpressed status and classical reported clinical, pathologic and prognostic factors (i.e. age, gender, family history, dietary and lifestyle habits (smoking, alcohol consumption, antioxidants, fatty foods and BMI), disease’s stages, GERD, *Helicobacter pylori* infections, pernicious anemia, presence of metastasis and treatment modalities). This is in contrast to available literature, where higher rates of HER2-positivity were found among the male gender, older ages (compared to the lowest age percentiles), intestinal-type tumors, metastasis and advanced tumor stages, and prognosis, among other factors [19, 28]. Studies have also previously described the influence of different risk factors on the incidence of GC, namely gender [6] socioeconomic status [7], dietary factors such as salt intake and tobacco, coffee and alcohol consumption [8–10], *Helicobacter pylori* infection [9, 14, 15], and gastric surgery [16]. A small study conducted has identified the need to target such risk factors, namely weight, physical activity and healthy diet with limited alcohol consumption, in public health interventions in order to reduce the risk of GC in the Lebanese population [33].

We otherwise report a significant correlation between HER2 overexpression and PS. This indicator is a simple clinical tool that can act as a surrogate marker for poor prognosis. Evidence shows that PS can independently reflect survival in advanced GC [34, 35]. Consistently, a meta-analysis of available literature identified a clear link between HER2-positivity and prognosis in GC [19], which could justify our findings seeing as both HER2 positivity and PS are associated with patient prognosis.

The incidence of *Helicobacter pylori* infections in patients diagnosed with GC in our study was 12%. This rate is substantially lower than those reported in other GC studies, where the incidence of *Helicobacter pylori* infection can be as high as 94.40% [15]. *Helicobacter pylori* infection is clearly described as a risk factor for GC *via* mechanisms that not only include the development and progression of chronic gastritis and gastric tumors, but also the compromise of immunotherapy efficacy [36–39]. Globally, close to 90% of non-cardia GC cases are attributable to *Helicobacter pylori* [14]. The risk of *Helicobacter pylori* infections extends to both cardia and non-cardia GC patients in high-risk settings [15]. This notable discrepancy between the incidence of *Helicobacter pylori* infections in our study and relevant literature brings into question the etiology and pathogenesis of GC in Lebanon, which remains unclear and should be explored.

GC is a highly heterogenous disease and caution is advised when assessing HER2 status, particularly when determined with IHC [40]. Several protocols were placed to promote reproducibility of HER2 testing in GC in light of the technical, tumor-related, sample-related and interpretation-related issues faced in this context. While ISH might still be considered the gold standard of HER2 testing, IHC was shown to have higher diagnostic accuracy [41]. The agreement between IHC and ISH results was found to be significantly substantial in this study. IHC can be considered as a good screening test in our population given that plotting ROC gave a good discrimination of the IHC test (AUC = 0.817; *p*-value = 0.01). A systematic review and meta-analysis of available evidence also showed good concordance between HER2 IHC and ISH in GC [41]. To note that this concordance was limited to HER2 IHC score 0/1 + or 3 + , suggesting the need for more detailed criteria in score 2 + [41]. Regardless of the agreement between ISH and IHC testing, one study noted that clinicians might consider conducting both IHC and ISH due to the improbability of consistent HER2 staining and laboratory results in the same community [42].

One limitation of the present study is the possibility of a classification bias with self-reported risk factors. As such, smoking, alcohol and fatty foods consumption could be underestimated. In parallel, the main strength point of the study is that HER2-neu status was tested according to a standardized protocol. Also, the study sample was chosen from nine centers in Lebanon and the patients were enrolled from all the Lebanese governorates with similar distribution to Lebanese population. In addition, we believe that these centers caught the majority of newly diagnosed cases of GC. Therefore, the results can be generalized to the Lebanese population.

## Conclusions

To conclude and to the best of our knowledge, the present study is the first nationally representative study to provide data on the prevalence of HER2-neu over expression in newly diagnosed early or metastatic GC patients in Lebanon (21%). The study demonstrated a significantly substantial level of agreement between the ICH and ISH results. Such information is essential for the management of GC in the country as it allows to identify candidates who stand to achieve clinical benefit from anti-HER2-neu therapy and to avoid unwarranted treatment of those unlikely to respond.

## Data Availability

The datasets used and/or analysed during the current study are available from the corresponding author on reasonable request.

## Abbreviations

AUC: Area Under the Curve
BMI: Body Mass Index
CEA: Carcinoembryonic Antigen
CI: Confidence Interval
ECOG: Eastern Cooperative Oncology Group
FISH: Fluorescence In-situ hybridization
GC: Gastric Cancer
GERD: Gastroesophageal Reflux Disease
GPP: Good Pharmacoepidemiology Practice
HER2-neu (aka HER2): Human Epidermal Growth Factor Receptor 2
HNPCC: Hereditary Non-Polyposis Colorectal Cancer
HR: Hazard Ratio
IHC: Immunohistochemistry
ISH: In-situ hybridization
NGS: Next-Generation Sequencing
PS: Performance Status
ROC: Receiver Operating Characteristic
TNM: Tumor, Node, Metastasis
